# Cross-testing of major molecular markers indicates distinct pathways of tumorigenesis in gastric adenocarcinomas and synchronous gastrointestinal stromal tumors

**DOI:** 10.1038/s41598-020-78232-2

**Published:** 2020-12-17

**Authors:** Éva Kocsmár, Ildikó Kocsmár, Luca Szalai, Gábor Lendvai, Attila Szijártó, Zsuzsa Schaff, András Kiss, Ilona Kovalszky, Gergő Papp, Gábor Lotz

**Affiliations:** 1 2nd Department of Pathology, Semmelweis University, Üllői str. 93, 1091 Budapest, Hungary; 2 1st Department of Surgery, Semmelweis University, Budapest, Hungary; 3 1st Department of Pathology and Experimental Cancer Research, Semmelweis University, Budapest, Hungary

**Keywords:** Gastric cancer, Gastrointestinal cancer, Oncogenesis, Cancer epidemiology, Cancer genetics

## Abstract

Small subtype of the gastrointestinal stromal tumor (micro-GIST, MG) is usually asymptomatic and is frequently found incidentally in association with gastric adenocarcinoma (GAC). The background of this coincidence is still an open question. This study comprehensively characterized nine MGs and GACs present in the same surgical specimen by cross-testing the markers of the major pathogenetic pathways of both tumor types. All of the MGs were immunohistochemically positive for CD117/KIT, CD34, and DOG1. DOG1 was also detected in four GACs. Four MGs carried mutations in c-KIT (exons 9, 11, and 13) and two cases in PDGFRα (exon 18). None of the GACs carried activating mutations in c-KIT or PDGFRα. MMR immunopanel identified one GAC as microsatellite unstable tumor. No EBV-positive tumor was found. According to the TCGA molecular classification, one GAC was categorized in the MSI subgroup, three GACs in the genomically stable subgroup, and the rest into the chromosomal instability subgroup. Although a common carcinogenic effect cannot be ruled out, our data suggest a distinct molecular background in the evolvement of the synchronous MGs and GACs. The presence of a MG in gastric resection specimens may be indicative of the development of synchronous malignant tumors in or outside the stomach.

## Introduction

Gastrointestinal stromal tumors (GISTs) arise from the interstitial cells of Cajal and can show either benign or malignant biological behavior^1,2^. Whilst small, indolent variant of GIST (GIST tumorlet, microscopic GIST, micro-GIST, MG) was commonly found to be up to 10 mm in size^3,4^, others defined it as small GISTs with a maximum diameter of 20 mm^5^. These macroscopically well-circumscribed lesions more frequently show spindle-cell morphology with a lower number of mitotic cells than the larger variants^6^. MGs are frequently found incidentally in the wall of the stomach during surgery or pathological examination of gastric resection specimens^7,8^.

Many case series were published in the last years, mostly from geographic areas having a high prevalence of gastric cancer^9,10^. In the Central European region, however, the general incidence of MGs in gastric resection specimens is less known.

Major part of GISTs are arising from mutations in the proto-oncogenic c-KIT receptor tyrosine kinase or intragenic activation mutation in the receptor tyrosine kinase domain of the platelet-derived growth factor receptor α (PDGFRα)^1,11^. Interestingly, in other published MG case series, these mutations were present in the early-stage/low-risk tumors, supporting these as early events in the tumorigenesis^5,6^.

In 2014, The Cancer Genome Atlas (TCGA) project provided a novel molecular classification of gastric cancer by introducing subgroups of carrying Epstein–Barr-Virus (EBV)-positivity, microsatellite instability (MSI), genomical stablity (GS) and chromosomal instability (CIN)^12^. The identified molecular subtypes are mutually exclusive and reveal the carcinogenetic pathway of the analyzed gastric cancer.

Since GISTs were identified as a separate entity in the ’80s, several issues have remained unanswered. The rising number of diagnosed MGs has drawn the attention to its synchronous presentation with other gastrointestinal malignancies, especially with gastric adenocarcinomas^3,9,13–16^. In order to explore this phenomenon, we comprehensively characterized gastric adenocarcinomas with coincidental MGs from the epidemiology of its synchronous presence to the analysis of the major molecular pathways of both tumors. To identify or exclude possible common molecular mechanisms in these neoplasms, known factors of tumor development of both the GISTs and adenocarcinomas (c-KIT/PDGFRα mutations, EBV/MSI status) were cross-tested on both tumor types.

## Results

### Cohort characteristics

The study enrolled 1027 patients that underwent surgical resection of the stomach between 2002 and 2018. In this period, 665 gastric adenocarcinomas were diagnosed, including 10 cases with additional MG. In the case of one patient, the available FFPE material was insufficient for further investigations therefore the present molecular analysis was conducted only on 9 cases having gastric adenocarcinoma and MG synchronously. The selection of the patients is shown in Supplementary Fig. S1 online. Detailed clinicopathological data of the synchronous tumors are shown in Table 1.

**Table 1:** Cohort characteristics and clinicopathological features of the tumors.

Case	Tumor	Site	Size (cm)	Gross appearance	Grade	pTNM	Age, sex	Gastrectomy	Other malignancy
1	GAC	Cardia	6.5	Infiltrative	G3	T2NxM0	61,F	Proximal	
	MG	Duodenum	2	Extraluminal	G1	T1N0M0			
2	GAC	Antrum	2	Ulcerative	G2	T1N0M0	70,M	Distal	
	MG	Corpus	0.7	Extraluminal	G1	T1N0M0			
3	GAC	Antrum	5	Ulcer, everted edge	G3	T2bN0M0	69,M	Distal	Lung cancer
	MG	Corpus	0.4	Extraluminal	G1	T1N0M0			
4	GAC	Cardia	6	Infiltrative	G3	T3N3M0	83,M	Proximal	
	MG	Duodenum	1.5	Extraluminal	G1	T1N0M0			
5	GAC	Corpus	2	Infiltrative	G3	T1N0M0	63,M	Total	
	MG	Corpus	0.2	Extraluminal	G1	T1N0M0			
6	GAC	Corpus	5	Ulcer, everted edge	G3	T2bN1M0	76,M	Proximal	
	MG	Corpus	0.3	Extraluminal	G1	T1N0M0			
7	GAC	Cardia	1	Villosus	G2	T1bN0M0	73,M	Total	
	MG	Corpus	0.5	Extraluminal	G1	T1N0M0			
8	GAC	Cardia	1.5	Infiltrative	G2	T3N1M0	78,M	Total	Oral cancer
	MG	Corpus	0.3	Extraluminal	G1	T1N0M0			
9	GAC	Antrum	3	Ulcer, everted edge	G2	T4aN2M0	75,M	Total	
	MG	Corpus	1	Extraluminal	G1	T1N0M0			

*GAC* gastric adenocarcinoma, *MG* microscopic gastrointestinal stromal tumor, *F* female, *M* male.

The general rate of MGs in patients operated due to gastric adenocarcinoma was 1.5% (10 out of 665 cases). In 50% of these patients, the small mesenchymal tumor was recognized during the surgery. In two of these five cases, the MG was located in the proximal area of the duodenum and not in the stomach. In the rest of the cases, MG was diagnosed only during the histopathological examination of the surgical specimens. Accordingly, prevalence of the incidental MGs was found to be low (0.97%, 10/1027 in all gastrectomy specimens; 1.5%, 10/665 next to gastric adenocarcinomas) in our study.

**Figure 1:**
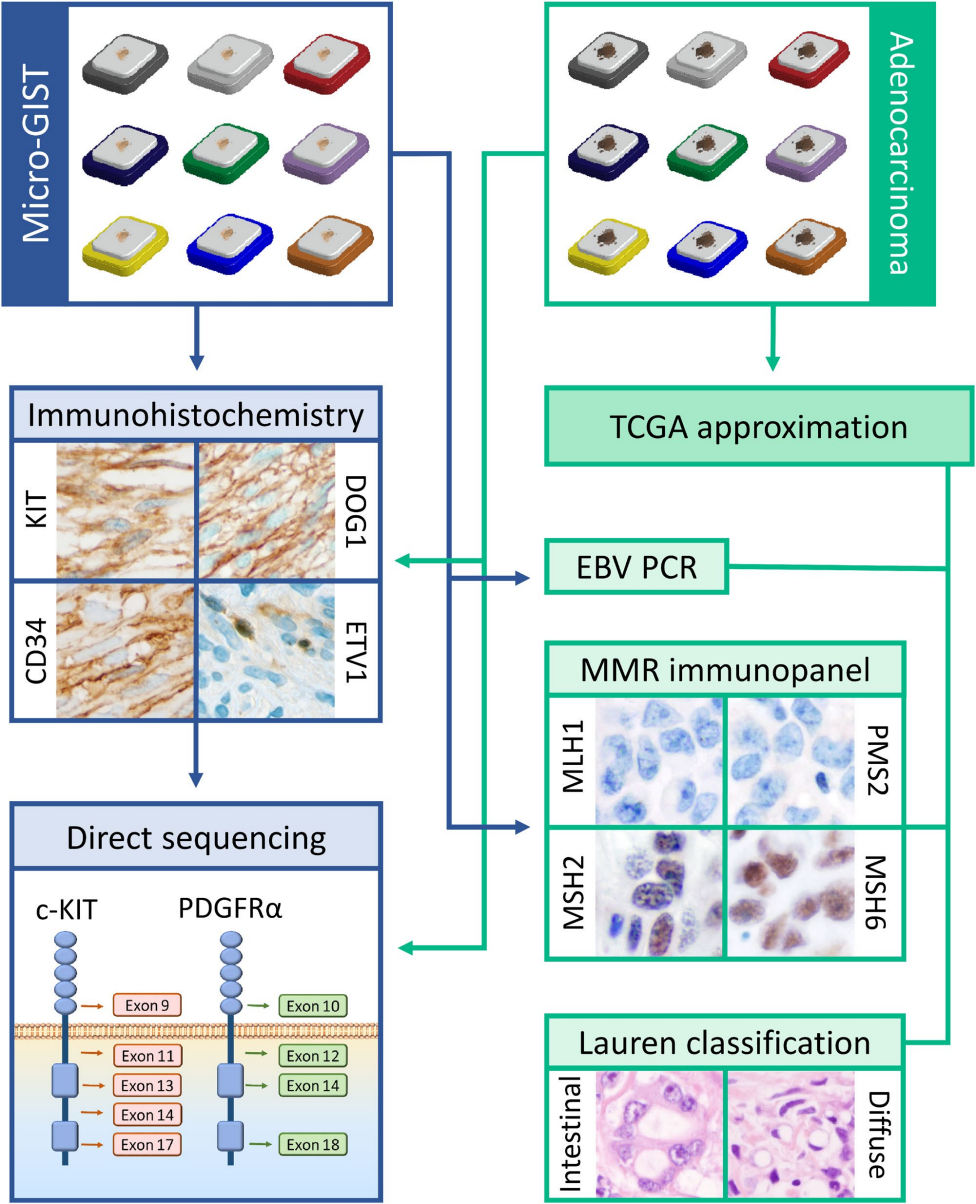
Workflow of the study. Cross-testing the markers of the major pathogenetic pathways of both micro-GISTs and gastric adenocarcinomas. *micro-GIST* microscopic gastrointestinal stromal tumor, *MMR* mismatch repair, *EBV* Epstein–Barr virus, *PCR* polymerase chain reaction, *TCGA* The Cancer Genome Atlas Project, *PDGFRα* platelet-derived growth factor-α, *DOG1* discovered on GIST-1, *ETV1* ETS translocation variant 1.

The mean age of the patients was 72 years (ranging from 61 to 83 years). Gender distribution was unbalanced in the study cohort (eight males, one female). The mean diameter of the MGs was 0.77 cm. According to the NIH (Fletcher) prognostic classification, all MGs belonged to the very low-risk group. Two of the nine patients had a history of a prior malignant tumor in their anamnestic data. The entire workflow is summarized in Fig. 1.

### Analysis of GIST predictors on both tumor types

**Figure 2:**
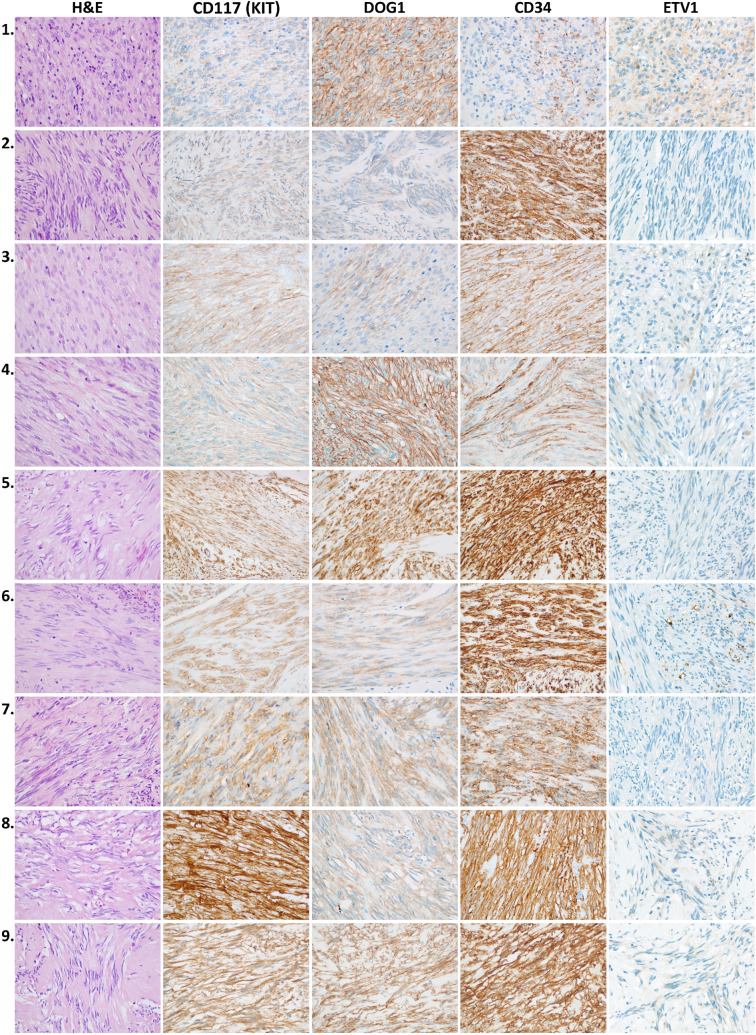
Immunohistochemical analysis of micro-GIST samples of the nine cases. *H&E* hematoxylin and eosin, *CD* cluster of differentiation, *DOG1* discovered on GIST-1. Original magnification: 600 ×.

All of the MGs displayed spindle cell morphology and showed positive immunostaining with CD117, CD34, and DOG1 (Fig. 2).

All adenocarcinomas were negative for CD117 and CD34, whereas positive DOG1 immunostaining was detected in four cases. C-KIT mutation was found in four GISTs, but no mutation was present in the synchronous adenocarcinomas. Two of the MGs carried mutation in exon 18 of the PDGFRα and one additional case had a benign single nucleotid variant in the PDGFRα exon 18 (V824V). This silent mutation was present in the corresponding adenocarcinoma sample too but none of the adenocarcinomas carried gain-of-function mutations in the investigated PDGFRα exons. Detailed results are shown in Table 2.

**Table 2:** Immunohistochemical analyses of GIST markers and c-KIT/PDGFRα mutational status of the tumors.

Case	Tumor	Immunohistochemistry				Sequencing		
		KIT	DOG-1	CD34	ETV1	c-KIT	PDGFRα	Mutation
1	GAC	**−**	**+/−**	**−**	**+/−**	WT	exon 18, SNV	p.V824V
	MG	**+**	**+++**	**+**	**+**	exon 9	exon 18, SNV	p.Y503_F504insAY, p.V824V
2	GAC	**−**	**+/−**	**−**	**−**	WT	WT	WT
	MG	**+**	**+**	**+++**	**−**	WT	WT	WT
3	GAC	**−**	**−**	**−**	**+/−**	WT	WT	WT
	MG	**++**	**+**	**++**	**+/−**	WT	exon 18	p.I843_D846delIMHD
4	GAC	**−**	**−**	**−**	**−**	WT	WT	WT
	MG	**+**	**+++**	**++**	**+/−**	WT	WT	WT
5	GAC	**−**	**−**	**−**	**−**	WT	WT	WT
	MG	**+++**	**+++**	**+++**	**−**	WT	exon 18	p.D842V
6	GAC	**−**	**−**	**−**	**+/−**	WT	WT	WT
	MG	**++**	**+**	**+++**	**+**	WT	WT	WT
7	GAC	**−**	**−**	**−**	**−**	WT	WT	WT
	MG	**++**	**++**	**+++**	**−**	exon 11	WT	p.V569_L576del
8	GAC	**−**	**+/−**	**−**	**+/−**	WT	WT	WT
	MG	**+++**	**+**	**+++**	**+/−**	exon 11	WT	p.V569_Q575del
9	GAC	**−**	**+**	**−**	**+/−**	WT	WT	WT
	MG	**+++**	**+++**	**+++**	**+/−**	exon 13	WT	p.K642E

*GAC* gastric adenocarcinoma, *MG* microscopic gastrointestinal stromal tumor (micro-GIST), *DOG1* discovered on GIST-1, *ETV1* ETS translocation variant 1 protein, *PDGFRα* platelet-derived growth factor-α, *SNV* single nucleotid variant, *WT* wild type.

### ETV1 (ETS translocation variant 1) immunohistochemistry

In the muscular wall of the stomach, we have observed an ETV1 staining pattern identical with the localization of the KIT-positive interstitial cells of Cajal (ICCs) which served as internal positive control (see Supplementary Fig. S2 online). The subcellular localization of the ETV1 immunohistochemical signal was not completely identical with the previously described nuclear positivity but a nuclear/cytoplasmic staining. Moreover, both in the ETV1 positive gastric adenocarcinomas and GISTs, we have observed a prevalent cytoplasmic positivity and only a small proportion of the positive cells showed nuclear/cytoplasmic subcellular localization pattern. No tumor budding associated special expression pattern of the ETV1 was observed in the adenocarcinomas. The ETV1 expression level was low in general among both the adenocarcinomas and the synchronous small GISTs (Table 2 and Fig. 2).

### EBV, MSI status and classification of the cases into molecular subtypes of gastric cancer

Since the TCGA molecular subtypes are based on the evolving major pathogenetic pathways during tumor development (EBV-positive, MSI, GS and CIN tumors)^12^, we characterized our cases according to a simplified version of this classification, described recently by Yoon et al.^17^. None of the adenocarcinomas were positive for EBV, determined by PCR. One case was classified as an MSI tumor based on the simultaneous loss of the MLH1 and PMS2 protein expression detected by MMR IH. The rest of the cases were subdivided according to the Lauren classification: three cases with diffuse histology were categorized as GS and five cases with intestinal histology as CIN.

In the nine synchronous MGs; however, the PCR-based analysis of the EBV positivity and immunohistochemical analysis of the MMR proteins provided negative results in all cases.

Details are shown in Table 3.

**Table 3:** EBV and MRI status of the tumors and approximated TCGA subtypes.

Case	Tumor	MMR immunopanel				EBV PCR	Lauren classification	TCGA
		MSH2	MSH6	MLH1	PMS2			
1	GAC	**+**	**+**	**+**	**+**	**−**	Diffuse	GS
	MG	**+**	**+**	**+**	**+**	**−**	**−**	**−**
2	GAC	**+**	**+**	**+**	**+**	**−**	Intestinal	CIN
	MG	**+**	**+**	**+**	**+**	**−**	**−**	
3	GAC	**+**	**+**	**−**	**−**	**−**	Intestinal	MSI
	MG	**+**	**+**	**+**	**+**	**−**	**−**	
4	GAC	**+**	**+**	**+**	**+**	**−**	Diffuse	GS
	MG	**+**	**+**	**+**	**+**	**−**	**−**	
5	GAC	**+**	**+**	**+**	**+**	**−**	Diffuse	GS
	MG	**+**	**+**	**+**	**+**	**−**	**−**	
6	GAC	**+**	**+**	**+**	**+**	**−**	Intestinal	CIN
	MG	**+**	**+**	**+**	**+**	**−**	**−**	
7	GAC	**+**	**+**	**+**	**+**	**−**	Intestinal	CIN
	MG	**+**	**+**	**+**	**+**	**−**	**−**	
8	GAC	**+**	**+**	**+**	**+**	**−**	Intestinal	CIN
	MG	**+**	**+**	**+**	**+**	**−**	**−**	
9	GAC	**+**	**+**	**+**	**+**	**−**	Intestinal	CIN
	MG	**+**	**+**	**+**	**+**	**−**	**−**	

*GAC* gastric adenocarcinoma, *MG* microscopic gastrointestinal stromal tumor (micro-GIST), *MMR* mismatch repair, *EBV* Epstein–Barr virus, *PCR* polymerase chain reaction, *TCGA* The Cancer Genome Atlas Project, *GS* Genomically Stable, *CIN* chromosomal instability.

## Discussion

The background of the frequently occurred incidental MGs next to gastric adenocarcinomas is still an open question. This study comprehensively characterized 9 MGs and adenocarcinomas present in the same surgical resection specimen by analyzing the c-KIT/PDGFRα status and EBV/MSI status on both tumor types.

GISTs are rare tumors located throughout the gastrointestinal tract with an estimated incidence of 10–15 per million people per year^18^. Epidemiological data are largely limited to large-size, mostly malignant subtypes of GISTs while the small, indolent MGs are usually not included in the cancer registries. Nevertheless, the general occurrence of MG in gastric resection specimens varies between 0.5 and 35%, which is markedly higher in the studies focused on its incidence next to gastric adenocarcinomas^4,19^ compared to that in patients without upper gastrointestinal neoplasms^8,20^. In contrast, our study revealed a relatively low prevalence of incidental MGs (0.97%, 10/1027 in all gastrectomy specimens; 1.5%, 10/665 next to gastric adenocarcinomas) in Central Europe.

An outstanding gender imbalance was present in the study cohort with male predominance (90%, 9 males/1 female), similarly to other studies^5,9,10,14,21^. This could partly be explained by the phenomenon that higher male predominance is a characteristic feature of gastric adenocarcinoma as compared to GIST alone^22^. No gastric adenocarcinoma with distant metastasis was found in the present study cohort, corresponding to the results of a published case series^10^.

There is a predilection site for MGs in the upper region of the stomach (fundus and body). Concordantly with the literature, 7 MGs of our cohort (77.8%, 7/9) were present in the gastric body and two further tumors were located in the proximal area of the duodenum. Small intestinal localization of GISTs was described to be associated with bad prognosis^23^ and in line with this, these tumors were the largest ones (1.5 cm, 2 cm) in our series. All MGs displayed spindle cell morphology and were categorized as very low-risk tumors, similarly to other case series^4,5,10^.

By immunohistochemical analysis, all MGs showed diffuse positivity for the GIST markers CD117, CD34, and DOG1, in various degrees. In their analogous study, Luo et al. found similar results^10^. In other studies investigating synchronous and solitary GISTs, however, Liu et al. found a lower frequency of DOG1 immunopositivity, whereas Lin et al. observed lower CD117 and CD34 positivity rates in the synchronous tumors^14,24^. When performing the same analyses on the synchronous adenocarcinomas, all cases proved to be negative for CD117 and CD34. However, four adenocarcinomas showed weak to focally strong DOG1 expression. As a transmembrane protein, DOG1 forms a Ca^2+^-mediated chloride channel and it is present in normal tissues of several organs due to its important role in secretory, sensory and contractile functions^25^. Several studies have examined DOG1 positivity in non-GIST tumors^26–31^. To the best of our knowledge; however, DOG1 expression of gastric adenocarcinomas was only investigated in one publication, a case series with immunohistochemistry, where a positivity rate of 27.6% (8/29) was found in intestinal-type carcinomas and 23.1% (3/13) in diffuse-type gastric cancers^31^. Our results are in line with this as two intestinal-type and two diffuse-type gastric adenocarcinomas expressed the DOG1 in our cohort.

We have also investigated the immunohistochemical expression of the ETV1 in the synchronous GISTs and adenocarcinomas since it was described as a transcription factor responsible for the differentiation of the interstitial cells of Cajal and to cooperate with KIT to promote GIST tumorigenesis^32^. However, the ETV1 is not universally expressed in the GISTs but was more frequently found in the early, small tumors (over 60%)^33,34^. Interestingly, we found similar rate of positivity (6/9) among our cases but with very low ETV1 expression level in all the positive tumors which is a peculiarity among the small indolent GISTs. On the other hand, ETV1 overexpression was shown to contribute to the epithelial to mesenchymal transition (EMT) by the upregulation of Snail expression and to be associated with higher tumor grade and stage in gastric adenocarcinomas^35^. The number of the patients in our case series is unfortunately too small to draw similar conclusions. Even so, we have carefully examined the adenocarcinomas in order to reveal any tumor budding-related special ETV1 expression pattern as the EMT is a key feature of the tumor budding^36^. Accordingly, one can speculate that the ETV1 expression level should be lower in the budding tumor cells of the invasion front. However, in our ETV1 positive gastric adenocarcinomas, no difference was apparent in the ETV1 expression pattern between the invasion front and the more central tumor areas. Another possible mechanism that a gastric adenocarcinoma might trigger the proliferation of the ICCs by inducing an elevated ETV1 expression and hereby contribute to the development of a GIST. But we found a significantly reduced ETV1 expression in the tumor cells of synchronous GISTs in comparison with the ICCs of the adjacent gastric wall. This suggests an ETV1-independent tumorigenesis or subsequent activation of antagonistic processes such as the degradation of ETV1 by the tumor suppressor COP1^37^. A further notable finding that all the three ETV1-negative GISTs were associated with ETV1 negativity of the synchronous adenocarcinoma as well (with strong ETV1 expression of the ICCs as internal positive control). However, we used another ETV1 primary antibody than in most of the previous studies since that one is not available anymore. This might be a considerable limitation from the aspect of comparability since instead of the previously described nuclear positivity we observed a different subcellular localization pattern with nuclear/cytoplasmic positivity of the ICCs and prevalent cytoplasmic and scattered nuclear/cytoplasmic positivity in both the GISTs and adenocarcinomas.

Although c-KIT or PDGFRα mutations are frequently present in the early GISTs as well^5,10^, their prevalence is generally lower in MGs as compared to larger GISTs^38,39^. Luo and his colleagues published a lower mutation rate in synchronous cases in comparison to solitary GISTs^10^. Our results provide further support to this, since only six of our cases carried mutations in c-KIT or PDGFRα, whereas the rest three cases proved to be negative for all of the investigated exons. The most frequently mutated exon was the c-KIT exon 11, concordantly with other studies^5,6,10,13^. One mutation in the exon 18 of the PDGFRα (D842V) is reported to be associated with resistance to imatinib^11,40^. Furthermore, mutated c-KIT and PDGFRα have been suggested to be involved in the pathogenesis of certain non-GIST tumors as well^41,42^. However, our gastric adenocarcinomas turned out to be negative for gain-of-function mutations in all of these mutational analyses.

Our approximated TCGA subtyping of adenocarcinomas showed slight differences in the proportional frequency of the four subgroups when being compared to the previous TCGA study^12^. The EBV-positive and MSI subtypes were underrepresented in our case series as none of our cases were positive for EBV infection (vs. the 8.8% rate in the previous TCGA study) and only one (11.1%) dMMR positive adenocarcinoma was found (vs. 21.7% rate for MSI subtype in the previous TCGA study). On the one hand, however, the low number of cases in our study (9 vs. 295 cases of the previous TCGA study) and the consequently high variability of the proportional frequency between the subgroups can explain these discrepancies. On the other hand, limitations of the used approximation approach for the TCGA subtyping might contribute to the observed slight differences as well. Regardless of the possible inaccuracy, our analysis clearly showed that there is no significant correlation between the TCGA subgroups and the presence of adjacent synchronous MG.

The EBV-positive and MSI gastric adenocarcinomas were reported to be associated with better prognosis in several studies and these tumors showed a higher response rate to immune-checkpoint inhibitors in a clinical trial^43–49^. Even though we used precise and sensitive diagnostic tools, lower number of cases of these better-prognosis subtypes were detected in our study, which suggests that adenocarcinomas with synchronous tumor have usually worse overall survival than the expected average prognosis of gastric cancers. Accordingly, as the immune-checkpoint inhibitor therapies are becoming more and more widely available, underrepresentation of the immunogenic EBV-positive and MSI subtypes will further widen the prognostic gap between the adenocarcinomas with and without synchronous MG.

Regarding the concomitant MGs, all cases were microsatellite stable and negative for EBV infection. This together with the absence of c-KIT and PDGFRα mutations in the adenocarcinomas confirm that the major molecular pathways of tumor development are different in gastric adenocarcinomas and adjacent MGs. This is important for a better understanding of the pathogenetic background of synchronous occurrence of GISTs and malignant tumors, which seems to be a relatively frequent event.

Several studies reported high sporadic coincidence of GISTs and other neoplasms and described GISTs as “sentinel tumors”^13,21,50^. In the case of adjacent synchronous tumors, we speculate that the gastrointestinal adenocarcinoma provides a growth factor-rich microenvironment, which induces the development of GIST from the nearby Cajal cells. In the majority of the cases with high-risk GISTs; however, the synchronous malignancy was reported to be present in extra-gastric localization^13,51^. This indicates that non-gastrointestinal cancers and GISTs either have common carcinogenic induction or the occurrence of one of the tumor types facilitates the development of the other tumor in a way of “action at a distance” (e.g. by circulating cytokines and growth factors, dysregulated vegetative nervous system, etc.).

Moreover, there is an interesting finding indicating a further relationship between synchronous tumors. Even though the prevalence of GISTs is higher when gastric cancer is present, it seems that the average size of the GIST is smaller than that of the solitary form. In the case series published previously, all of the synchronously occurred GISTs are categorized as MGs with a maximum diameter of ≤ 20 mm^9,10^, or only a few millimeters larger in size^14,51^. Our results provide further support to this as the average diameter of the synchronous GISTs was 7.67 mm (2–20 mm), indicating that all of them are MGs, indeed. Interestingly, the average diameter of the MGs located more distant from the synchronous gastric adenocarcinoma (one of them is proximally while the other distally situated, see in Table 1) was larger than the diameter of MGs localized in the same region (proximal/proximal, distal/distal) like the adenocarcinoma (3.225 mm vs. 11.2 mm, respectively). These observations contradict the idea that locally accumulated growth factors or any other local factors have potential roles in contributing to the adenocarcinoma—MG synchronism, but raise the possibility that gastric cancer participates in the controlling of biological behavior of MGs^9^. It is also possible, however, that these small lesions adjacent to a surgically removed cancer are more easily detectable during the grossing, which may explain the overrepresentation of MGs in the synchronous tumors of gastric adenocarcinomas. Nevertheless, the frequent association of GISTs with other tumors (two of our patients had a history of a prior malignant tumor as well) draws the attention to the prognostic role of synchronous MGs in view of developing further malignancies as well.

To the best of our knowledge, our study characterized one of the largest case series of gastric adenocarcinomas with synchronous MGs and it is the only one which analyzed the background of this simultaneous appearance by taking the pathomechanism of both tumor types into consideration. Notwithstanding, the number of cases and the considered immunohistochemical and molecular markers may be less than to be able to draw more precise conclusions about the potential common tumorigenesis. Furthermore, the retrospective character of the study limited to examine the resection specimens in higher detail than the routine grossing process and to analyze more clinicopathological conditions. Therefore, more precise identification of the groups of patients with higher risk to develop synchronous tumors was not possible.

To conclude, our data further support that distinct mechanisms play roles in the development of synchronous GISTs and gastric adenocarcinomas, although a common carcinogenic effect cannot be ruled out. In light of our study, it is important to systematically look for associated MGs during a gastrectomy due to gastric cancer and during the grossing of its resection specimen. Regarding the possible “sentinel” role of the GISTs, however, it is also worth keeping in mind that malignant tumors of other primary sites can be associated with these mesenchymal tumors as well.

## Materials and methods

### Case selection procedure and ethical permission

During the study period (2002–2018), gastrectomy specimens containing gastric adenocarcinoma and synchronous MG were selected from the institutional archives of the 2nd Department of Pathology for this retrospective cross-sectional study. Detailed clinical data of the patients were collected from the electronic patient register of Semmelweis University. All cases with sufficient tumor content in the available formalin-fixed, paraffin-embedded (FFPE) tissue block(s) were included in the study (n = 9). The detailed case selection procedure is shown in Supplementary Fig. S1 online.

The study protocol was following the ethical guidelines of the 1975 Declaration of Helsinki and was approved by the Ethical Committee of the Semmelweis University, Budapest (#22/2019). Based on the current Hungarian law for the scientific research, contacting the patients in order to have their informed consent is basically not requested for the retrospective studies. According to this, the Ethical Committee of the Semmelweis University, Budapest has waived the informed consent procedure for the study.

### Routine histopathologic work-up and immunhistochemical analysis

Diagnostic work-up of gastrectomy specimens was performed according to a standardized protocol. Tissue samples were fixed with formalin and embedded in paraffin (FFPE). Tissue sections of 3 μm thickness were cut from the FFPE tissue blocks and stained with hematoxylin and eosin. For the routine diagnostic purpose of gastric adenocarcinomas and GISTs, immunohistochemical stainings were performed in the representative areas of the resection specimens when it was necessary for the diagnosis. The cases containing both tumor types synchronously were re-analyzed by an experienced histopathologist (GL) and detailed histologic features were recorded (see results). Stages of both tumors were classified according to the eighth edition of the UICC TNM classification of malignant tumors^52^. For prognostic stratification of GIST tumors, the NIH standard was used proposed by Fletcher et al. in 2002^53^. GISTs up to 20 mm were considered as MG in this study, based on the definition by Anderson et al.^5^.

The histological subtype of gastric adenocarcinomas was classified according to the Lauren classification.

For the analyses, patients were randomly numbered from 1 to 9 and the two distinct tumors were labeled as 1GAC (gastric adenocarcinoma of Patient 1) and 1MG (micro-GIST of Patient 1). All analyses were performed on both the GAC and the MG components of resection specimens. For the immunohistochemical (IH) analyses, 4 µm thick tissue sections were cut from the FFPE tissue blocks and mounted on a coated glass slide. Stainings were performed on a Ventana Benchmark Ultra automated IH staining system using standard protocol including steps of heat pretreatment with pH = 9 Cell Conditioning 1 and signal development with UltraView Universal DAB Detection Kit (Ventana Medical Systems, Inc, Tucson, AZ, USA). Hematoxylin was used for counterstaining.

Both tumor types were tested with GIST immunomarkers, including anti-CD117/c-KT (EP10, BioCare, 1:300, monoclonal rabbit), anti-CD34 (QBEnd-10, Bio SB, 1:500, monoclonal mouse) and anti-DOG1 (NCL-L-DOG-1, Leica, 1:100, monoclonal mouse) as well as by anti-ETV1 (PA5-41484, Invitrogen, 1:150, polyclonal rabbit) primary antibody. The intensity of these IH stainings was ranked as follows: − (negative), + /− (focal week), + (weak), ++ (moderate), and +++ (strong).

For detection of Mismatch Repair (MMR) protein status, a panel of four ready-to-use (RTU) primary antibodies was used: anti-MLH1 (clone: M1, monoclonal mouse), anti-MSH2 (clone: G219-1129, monoclonal mouse), MSH6 (clone: SP93, monoclonal rabbit) and PMS2 (clone: A16-4, monoclonal mouse) (Ventana Medical Systems, Inc., Tucson, AZ). Epithelial cells of the intestinal crypts were used as positive controls. When a loss of any of these proteins was detected (corresponding to the significant reduction in IH staining intensity of the specific nuclear positivity), the tumor was referred to as mismatch repair deficient (dMMR) and classified into the MSI subgroup. The primarily affected protein of the heterodimer pairs was determined by the following scheme: MLH1-/PMS2- → MLH1; MLH1 + /PMS2- → PMS2; MSH2-/MSH6- → MSH2; MSH2 + /MSH6- → MSH6.

### DNA extraction and molecular analyses

#### DNA extraction

From FFPE tissue blocks, three sections of 10 μm were cut and areas containing at least 80% of the tumor cells were manually macrodissected using a marked hematoxylin-eosin-stained slide from the corresponding tissue block as a guide. To reduce cross-contamination between the cases, the microtome was cleaned with non-denaturated absolute ethanol in-between each tissue-block sectioning. Following deparaffinization, DNA extraction was performed using the Roche High Pure FFPET DNA Isolation Kit (Roche GmbH, Mannheim, Germany) following the manufacturer’s instructions. The quantity of the extracted DNA was measured by NanoDrop 1000 Spectrophotometer (Life Technologies of Thermo Fischer Scientific, Waltham, MA, USA).

#### c-KIT and PDGFRα polymerase chain reaction and sequencing analysis

Exons 9, 11, 13, 14 and 17 of c-KIT and exons 10, 12, 14, and 18 of PDGFRα were screened for mutations using amplification of genomic DNA by polymerase chain reaction (PCR) followed by direct sequencing of PCR products. Supplementary Table S1 provides the details for the primers. A reaction volume of 20 μL was used, containing 10 μL of 2 × ImmoMix Red (Bioline Reagents Ltd., London, UK), 20 pmol of each primer, and 1 μL of DNA template solution. PCR products were analyzed on 2% DNA-quality agarose gels and were visualized using an Invitrogen iBright FL1000 Imaging System (Thermo Fisher Scientific, Waltham MA, USA). Direct sequencing was performed with BigDye Terminator V3.1 Cycle Sequencing Kit from both directions using an Applied Biosystems 3500 Series Genetic Analyzer (Applied Biosystems, Foster City, CA) according to the manufacturer’s instructions. The sequence of an individual exon was compared with the corresponding gene sequence available in the National Center for Biotechnology Information GenBank database: http://www.ncbi.nlm.nih.gov.

#### EBV PCR analysis

Detection of EBV DNA by PCR analysis was carried out under standard conditions. The sequences of the applied primers are listed in Supplementary Table S1. A typical 25-μl amplification reaction mixture contained 12.5 μL of 2 × ImmoMix Red (Bioline Reagents Ltd., London, UK), the two primers (20 pmol each), and 1 μL of DNA solution. Each of the 40 PCR cycles included a denaturation step at 95 °C for 20 s, followed by annealing at 55 °C for 30 s and an extension at 72 °C for 50 s. PCR amplicons were analyzed by electrophoresis using a 2% agarose gel and visualized by an Invitrogen iBright FL1000 Imaging System (Thermo Fisher Scientific, Waltham, MA, USA).

### EBV, MSI status and classification of the cases into molecular subtypes of gastric cancer

Since TCGA classifies gastric cancers into molecular subtypes based on major pathogenetic pathways (EBV-positive, MSI, GS, and CIN tumors)^12^, we characterized our cases according to this classification. We used a simplified, dichotomous algorithm described in recent publications^17,43^. Based on a recently published study from Yoon and his colleagues^17^, an approximated reproduction of the TCGA classification is achievable by an algorithm starting with the analysis of the EBV positivity, following by the investigation of the MSI status. Finally, the GS and CIN tumors are separated by the histological subtypes according to the Lauren classification (GS: diffuse, CIN: intestinal type).

## Supplementary information

Supplementary Table S1.

Supplementary Figure S1.

Supplementary Figure S2.
