# Supplementary material for: Cross-testing of major molecular markers indicates distinct pathways of tumorigenesis in gastric adenocarcinomas and synchronous gastrointestinal stromal tumors
Source: Sci Rep. 2020 Dec 17;10:22212. doi: 10.1038/s41598-020-78232-2 (PMC7747598; doi:10.1038/s41598-020-78232-2)
Supplement: Supplementary file 1 — Supplementary Table S1. [file 41598_2020_78232_MOESM1_ESM.docx]

**Cross-testing of major molecular markers indicates distinct pathways of tumorigenesis in gastric adenocarcinomas and synchronous gastrointestinal stromal tumors**

Éva Kocsmár, Ildikó Kocsmár, Luca Szalai, Gábor Lendvai, Attila Szijártó, Zsuzsa Schaff, András Kiss, Ilona Kovalszky, Gergő Papp, Gábor Lotz

**Supplementary Table S1. The details of primers used in the study**

| **Target** | **Exon** | **Primer name** | **Primer Sequence 5’-3’** | **Amplicon (bp)** |
| --- | --- | --- | --- | --- |
| KIT | 9 | KIT9-F | GCCACATCCCAAGTGTTTTATG | 310 |
|  |  | KIT9-R | GAGCCTAAACATCCCCTTAAATTG |  |
| KIT | 11 | KIT11-F | CCAGAGTGCTCTAATGACTG | 223 |
|  |  | KIT11-R | AGCCCCTGTTTCATACTGAC |  |
| KIT | 13 | KIT13-F | CTTGACATCAGTTTGCCAGTTGT | 203 |
|  |  | KIT13-R | GACAGACAATAAAAGGCAGCTTG |  |
| KIT | 17 | KIT17-F | TGGTTTTCTTTTCTCCTCCAA | 184 |
|  |  | KIT17-R | GCAGGACTGTCAAGCAGAGA |  |
| KIT | 14 | KIT14-F | GTCTGATCCACTGAAGCTG | 319 |
|  |  | KIT14-R | ACCCCATGAACTGCCTGTC |  |
| PDGFRα | 10 | PDGFRA10-F | GGCCCTATACTTAGGCCCTTTT | 251 |
|  |  | PDGFRA10-R | TGTCCTGACTGTTGAGGAACT |  |
| PDGFRα | 12 | PDGFRA12-F | CTCTGGTGCACTGGGACTTT | 233 |
|  |  | PDGFRA12-R | GCAAGGGAAAAGGGAGTCTT |  |
| PDGFRα | 14 | PDGFRA14-F | TCTGAGAACAGGAAGTTGGTAGC | 208 |
|  |  | PDGFRA14-R | CCAGTGAAAATCCTCACTCCA |  |
| PDGFRα | 18 | PDGFRA18-F | CTTGCAGGGGTGATGCTATT | 230 |
|  |  | PDGFRA18-R | AGAAGCAACACCTGACTTTAGAGATTA |  |
| EBV | - | BamH1-K -F | CCGGTGTGTTCGTATATGGAG | 106 |
|  |  | BamH1-K -R | GGGAGACGACTCAATGGTGTA |  |
